# Reassessment of carotid intima-media thickness by standard deviation score in children and adolescents after Kawasaki disease

**DOI:** 10.1186/s40064-015-1275-1

**Published:** 2015-09-04

**Authors:** Nobutaka Noto, Masataka Kato, Yuriko Abe, Hiroshi Kamiyama, Kensuke Karasawa, Mamoru Ayusawa, Shori Takahashi

**Affiliations:** Department of Pediatrics and Child Health, Nihon University School of Medicine, 30-1 Oyaguchikami-machi, Itabashi-ku, Tokyo, 173-8610 Japan; Noto Children’s Clinic, 4-12-6 Heiwadai, Nerima-ku, Tokyo, 179-0083 Japan

**Keywords:** Kawasaki disease (KD), Carotid intima-media thickness (CIMT), Standard deviation score (SDS), Subclinical atherosclerosis

## Abstract

Previous studies that used carotid ultrasound have been largely conflicting in regards to whether or not patients after Kawasaki disease (KD) have a greater carotid intima-media thickness (CIMT) than controls. To test the hypothesis that there are significant differences between the values of CIMT expressed as absolute values and standard deviation scores (SDS) in children and adolescents after KD and controls, we reviewed 12 published articles regarding CIMT on KD patients and controls. The mean ± SD of absolute CIMT (mm) in the KD patients and controls obtained from each article was transformed to SDS (CIMT-SDS) using age-specific reference values established by Jourdan et al. (J: n = 247) and our own data (N: n = 175), and the results among these 12 articles were compared between the two groups and the references for comparison of racial disparities. There were no significant differences in mean absolute CIMT and mean CIMT-SDS for J between KD patients and controls (0.46 ± 0.06 mm vs. 0.44 ± 0.04 mm, p = 0.133, and 1.80 ± 0.84 vs. 1.25 ± 0.12, p = 0.159, respectively). However, there were significant differences in mean CIMT-SDS for N between KD patients and controls (0.60 ± 0.71 vs. 0.01 ± 0.65, p = 0.042). When we assessed the nine articles on Asian subjects, the difference of CIMT-SDS between the two groups was invariably significant only for N (p = 0.015). Compared with the reference values, CIMT-SDS of controls was within the normal range at a rate of 41.6 % for J and 91.6 % for N. These results indicate that age- and race-specific reference values for CIMT are mandatory for performing accurate assessment of the vascular status in healthy children and adolescents, particularly in those after KD considered at increased long-term cardiovascular risk.

## Background

Several studies showed that carotid intima-media thickness (CIMT), a surrogate of atherosclerotic vessel wall changes, is sensitive to risk intervention and constitutes a reliable indicator of clinical outcome in adult subjects, even in a pediatric cohort (O’Leary et al. 1999; Jarvisalo et al. 2001). Moreover, these changes in the arterial wall occur earlier in the carotid arteries than in the coronary circulation, making the carotid arteries an ideal site for the detection of premature atherosclerotic disease (Bland et al. 1986).

Kawasaki disease (KD) is characterized by severe systemic vasculitis of unknown etiology occurring in infants and young children (Kawasaki 1967). In the convalescent stage, compared with that in controls, the carotid arterial wall has been reported to have higher CIMT and lower distensibility in KD patients with persistent coronary artery lesions (CALs) (Noto et al. 2001, 2009; Cheung et al. 2007; Della Pozza et al. 2007; Cheung et al. 2008). Moreover, the CIMT, expressed as both unadjusted dimension and standard deviation scores (SDS), was greater among patients with KD than in control subjects; within the KD group, patients with CALs had greater CIMT than those without CALs (Meena et al. 2013). However, some investigators have found no difference in CIMT between KD patients and controls (Kadono et al. 2005; Ikemoto et al. 2005; Gupta-Malhotra et al. 2009; Lee et al. 2009; Selamet Tierney et al. 2013; Ishikawa and Iwasahima 2013).

In view of such conflicting clinical data, we wished to investigate directly the values of CIMT derived from the articles published to date. The methodology for the assessment of CIMT is of particular interest because the use of reference values on CIMT by LMS methods for childhood and adolescence is limited when comparing patients after KD and controls (Meena et al. 2013). We speculated that there are significant differences between the values of absolute CIMT and those of SDS transformed by LMS methods (CIMT-SDS) in children and adolescents after KD and controls. To test this hypothesis, we reviewed previously published articles regarding CIMT in KD patients and controls.

## Methods

### Search

We attempted to find all publications describing CIMT in KD patients and controls. Reports on potentially eligible studies were retrieved via electronic databases (MEDLINE, EMBASE, CENTRAL). The following (combination of) medical subject heading and free text key words were used: “Kawasaki disease”, “Carotid intima-media thickness”, “Carotid arteries”, “Subclinical atherosclerosis”, and “Premature atherosclerosis”. Additional studies were sought by a manual research through reference lists of relevant publications, recent reviews, and editorials and through personal communication with experts in this field.

### Study selection and data extraction

Two reviewers determined the eligibility of the retrieved studies independently, according to predetermined criteria. The following criteria were accepted for inclusion in this study: (1) subjects with a diagnosis of KD, (2) an interval from initial onset of illness of ≥1 year, (3) KD patients and control subjects who underwent carotid ultrasound according to the Mannheim CIMT consensus, (4) mean CIMT measurements at least in triplicate by the far wall of either right or left common carotid artery (CCA), and (5) the use of either manual tracing with electronic calipers or a semiautomated border detection program for the assessment of CIMT. Subjects were excluded if they had potentially confounding coronary risk factors (CRFs) including dyslipidemia, diabetes mellitus, hypertension, smoking history, or a family history of premature coronary artery disease (CAD), and if they were in duplicate reports or preliminary reports of data later presented in full. Data of the selected articles were extracted by two reviewers independently. Differences in judgment by the reviewers were resolved by discussion and consensus.

### Data transformation and analysis

The LMS method is widely used for the description of pediatric anthropometric data, and allows the calculation of percentiles and accurately normalized SDS accounting for nonlinearity and a skewed distribution of the reference data set (Cole and Green 1992). The LMS method describes the distribution of the measurement Y by its median (M), the coefficient of variation (S), and a measure of skewness (L) required to transform the data to normality: SDS = {[Y/M(t)]^L(t)^ − 1}/[L(t) × S(t)]. Here, Y is the individual measurement and L, M, and S originate from the specific reference values for each age (t).

The absolute mean ± SD of CIMT in the KD patients and controls was transformed to SDS using age-specific reference values for the mean CIMT established using the LMS method by Jourdan et al. (2005) and our own data, and the results were compared between the KD patients and the controls and the two references for comparison of racial disparities. In this study, the mean age of the study population derived from each article was designated as the representative age for transformation.

Continuous variables were compared using unpaired *t* test or nonparametric Mann–Whitney *u* tests. Two-sided values of p < 0.05 were considered significant. All statistical analyses were performed using SPSS version 10.0 (SPSS Inc., Chicago, IL, USA).

### Reference values for CIMT

**Fig. 1:**
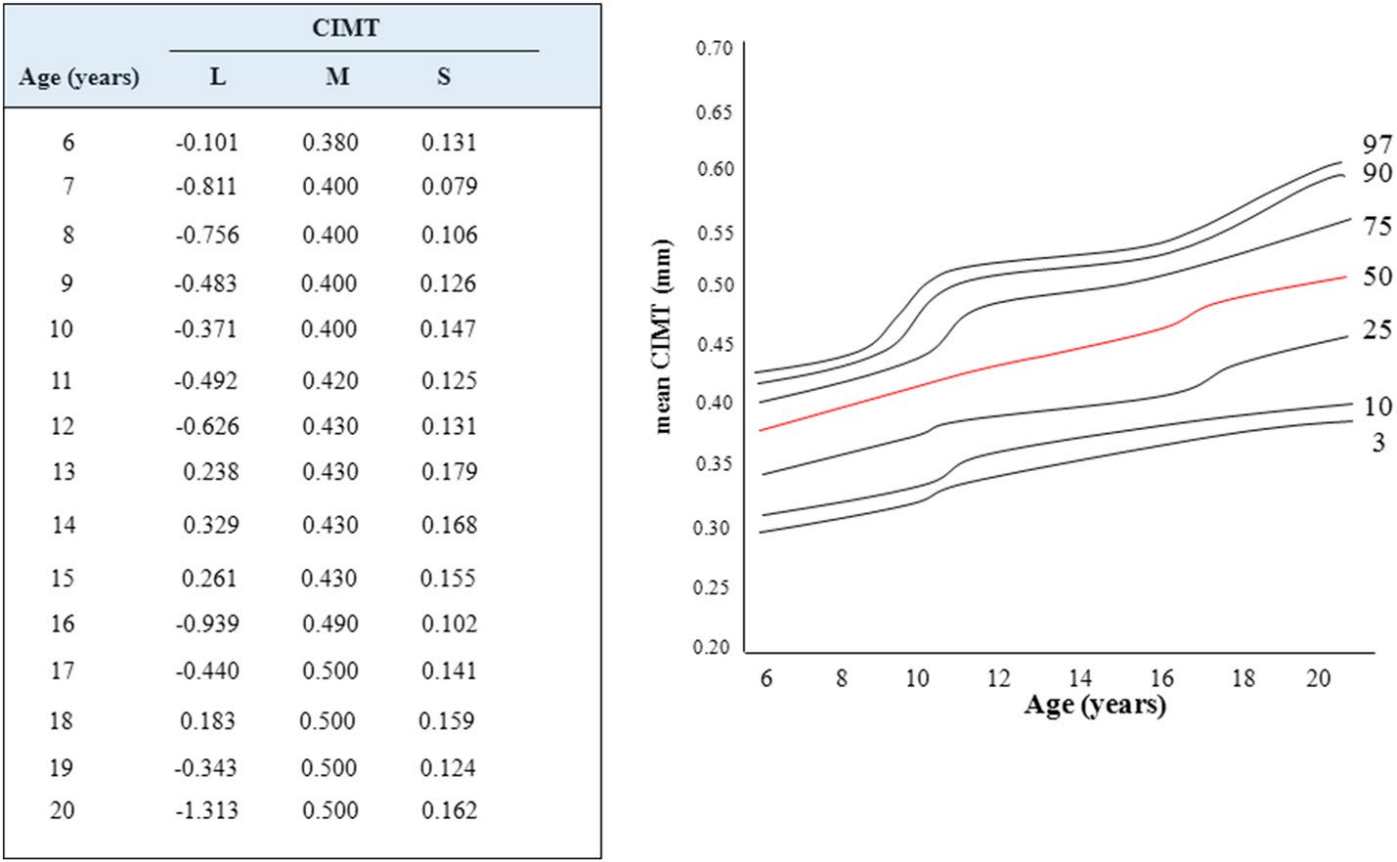
Age-specific reference values and percentile curve for CIMT. *CIMT* carotid intima-media thickness, *L* skewness coefficient, *M* median, *S* coefficient of variation. *Numbers* indicate 3rd, 10th, 25th, 50th, 75th, 90th, and 97th percentiles

Age-specific normative reference values for mean CIMT established by Jourdan et al. (J) consisted of 247 healthy Caucasian adolescents aged 10–20 years, as described previously (Jourdan et al. 2005). Meanwhile, our own reference values (N) consisted of 175 (male/female = 96/79) healthy Asian school-age subjects and their siblings aged 6–20 years who were free from obesity or hypertension (Fig. 1). None took medication or had blood sampling data. All were nonsmokers with no history of cardiac disease. Anthropometric data of the reference subjects, namely of height, body mass index, systolic blood pressure, diastolic blood pressure, and pulse pressure, were 156.9 ± 12.6 (cm), 19.5 ± 2.0 (kg/m^2^), 109.0 ± 10.6 (mmHg), 60.4 ± 8.9 (mmHg), and 47.8 ± 9.8 (mmHg), respectively.

LMS estimates were obtained for age, and percentile graphs were constructed from the L, M, and S data. In both of the references, mean CIMT measured from carotid ultrasound was obtained in a standard manner according to the Mannheim CIMT consensus, as published previously (Touboul et al. 2012). In brief, the CCAs were assessed 1–2 cm proximal of the bifurcation over a range of 1 cm of the far wall. CIMT was defined as the distance between the leading edge of the lumen-intima interface and the media-adventitia interface of the far wall measured during diastole.

The distribution of CIMT did not differ significantly between male and female subjects at any given age in both references. Although CIMT was significantly associated with age, height, body mass index, systolic blood pressure, and pulse pressure in both references, we confined the reference to exclusively the age-specific reference because of the limitation of extractable data from each article in this study. Interobserver and intraobserver variability studied in our laboratory with repeated recordings has shown coefficients of variance for mean CIMT of 7.3 and 5.0 %, respectively (Noto et al. 2001).

## Results

### Characteristics of the selected articles

Our search yielded 13 publications, which were all on case–control studies. One of these had to be excluded because it met 1 of the exclusion criteria. The characteristics of the 12 remaining studies, published from 2001 to 2013, are shown in (Table 1). The number of participants in the studies varied from 20 to 203. In total, 572 KD patients and 375 control subjects were assessed. The mean ages of the KD patients and controls were 13.1 ± 3.6 years (range 6.5–20.9 years) and 14.0 ± 3.7 years (range 7.9–21.3 years), respectively. About 65 % of the KD patients and 60 % of the controls were male. The mean interval from the initial onset of illness was 8.9 ± 5.2 years (range 2.6–18.6 years) in the KD patients. Except for the three articles with unknown treatments at the acute phase, about 85 % of the KD patients had been treated with intravenous immunoglobulin during the acute phase of illness.

**Table 1:** Characteristics of the 12 selected articles regarding CIMT of KD patients and controls

First author year (ref.)	KD patients					Age (years)	Male (%)	Follow-up (years)	Controls (n)	Age (years)	Measurements; site	Tracing	CIMT(KD) (mm)	CIMT(Cont) (mm)	p value	Remarks
	Int (n)	Ect (n)	Reg (n)	An (n)	Giat (n)											
Noto et al. (2001)	0	0	0	20	0	16.6 ± 4.1	66	9.8	20	16.3 ± 4.7	Mean; far wall CCA	Manual	0.53 ± 0.07	0.46 ± 0.05	<0.050	An vs. Cont
Kadono et al. (2005)	0	0	9	15	0	8.3 ± 4.1	54	5.8	41	10.7 ± 4.4	Mean; far wall CCA	Manual	0.45 ± 0.07	0.46 ± 0.06	0.544	Reg + An vs. Cont
Ikemoto et al. (2005)	31	16	0	8	10	12.7 ± 3.5	58	12	20	16.2 ± 4.0	Mean; far wall CCA	Manual	0.52 ± 0.05	0.50 ± 0.04	0.220	Giat vs. Cont
Cheung et al. (2007)	24	0	0	26	0	8.6 ± 2.8	66	7.4	22	9.5 ± 2.2	Mean; far wall CCA	Manual	0.41 ± 0.04	0.36 ± 0.04	<0.001	An vs. Cont
Della Pozza et al. (2007)	5	0	0	15	0	12.1 ± 4.7	60	4.1	28	12.0 ± 3.1	Mean; far wall CCA	Automated	0.44 ± 0.02	0.42 ± 0.01	<0.001	Int + An vs. Cont
Cheung et al. (2008)	19	0	6	26	0	13.4 ± 0.6	78	10.5	32	14.6 ± 0.6	Mean; far wall CCA	Automated	0.44 ± 0.02	0.42 ± 0.02	0.006	An vs. Cont
Noto et al. (2009)	0	0	0	35	0	20.5 ± 9.3	80	18.6	35	19.6 ± 7.2	Mean; far wall CCA	Manual	0.57 ± 0.15	0.47 ± 0.01	<0.001	An vs. Cont
Gupta-Malhotra et al. (2009)	19	0	9	0	0	20.9 ± 6.0	67	16.6	27	21.3 ± 7.5	Mean; far wall CCA	Manual	0.49 ± 0.07	0.48 ± 0.06	0.905	Int + Reg vs. Cont
Lee et al. (2009)	0	0	25	0	0	12.6 ± 2.0	ND	8	55	14.5 ± 0.7	Mean; far wall CCA	Manual	0.41 ± 0.19	0.50 ± 0.01	0.026	Reg vs. Cont
Meena et al. (2013)	17	0	9	1	0	8.2 ± 2.6	74	2.6	23	8.4 ± 2.9	Mean; far wall CCA	Manual	0.49 ± 0.07	0.41 ± 0.06	<0.001	Int + Reg + An vs. Cont
Selamet Tierney et al. (2013)	136	20	0	40	7	16.7 ± 4.2	60	11.6	50	17.5 ± 4.3	Mean; far wall CCA	Automated	0.44 ± 0.03	0.43 ± 0.03	0.420	Int + Ect + An + Giat vs. Cont
Ishikawa and Iwasahima (2013)	15	0	0	9	0	7.2 ± 2.3	58	3.3	22	7.9 ± 2.8	Mean; far wall CCA	Automated	0.45 ± 0.03	0.43 ± 0.04	0.906	Int vs. An vs. Cont

*An* coronary aneurysm, *Cont* controls, *CIMT* carotid intima-media thickness, *Ect* ectasia, *Giat* giant aneurysm, *Int* intact, *ND* not described, *Reg* regression

### Comparison of CIMT between KD patients and control subjects

**Fig. 2:**
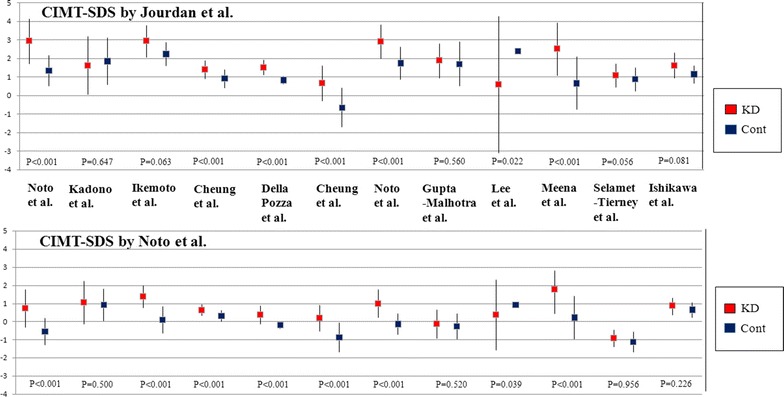
Difference of the values of CIMT-SDS between the two references in KD patients and controls among the 12 articles. *CIMT-SDS* standard deviation score of carotid intima-media thickness, *Cont* controls, *KD* Kawasaki disease patients, *p* p value

The absolute values of CIMT in the KD patients were significantly higher than those in the controls in 6 of 12 (50 %) articles. Likewise, CIMT-SDS transformed by J or N in the KD patients was significantly higher than in the controls in 6 of 12 (50.0 %) articles in J and 7 of 12 (58.3 %) articles in N. On the whole, there were no significant differences in mean absolute CIMT and mean CIMT-SDS transformed by J among the 12 articles between the two groups (0.46 ± 0.06 mm vs. 0.44 ± 0.04 mm, p = 0.133, and 1.80 ± 0.84 vs. 1.25 ± 0.12, p = 0.159, respectively). However, there were significant differences in mean CIMT-SDS for N among the 12 articles between the KD patients and the controls (0.60 ± 0.71 vs. 0.01 ± 0.65, p = 0.042). Compared with the two reference values, CIMT-SDS in the controls for N was significantly lower than that for J (p < 0.001). Therefore, CIMT-SDS or N in the controls was within the normal range of mean ± SD in 11 of 12 (91.6 %) articles in comparison with that for J in 5 of 12 (41.6 %) articles (Fig. 2). When we assessed the 9 articles on Asian subjects, the difference of CIMT-SDS between the KD patients and the controls was invariably significant only for N (p = 0.015). In contrast, when we assessed the 3 articles on mainly Caucasian subjects, there was no significant difference in CIMT-SDS between the KD patients and the controls in either J (p = 0.163) or N (p = 0.162).

## Discussion

Our results showed that CIMT-SDS among the 12 articles was significantly different between the KD patients and the controls despite the absolute CIMT not differing significantly between these two groups. Moreover, the normative reference values for CIMT applied in this study allowed comparison of the accurate vascular phenotypes of KD patients with those of healthy subjects.

Although it is well established that CIMT increases with age in adults, age-associated changes of CIMT in children have not been fully explored. As for the normative values of CIMT in children and adolescents among previously published papers, mean CIMT (mean ± SD) in the distal common carotid artery has been reported as 0.46 ± 0.06 mm for 30 healthy children aged 14 ± 2 years (Tonstad et al. 1996), 0.47 ± 0.04 mm for 80 healthy controls aged 8–18 years (Wiegman et al. 2004), 0.42 ± 0.04 mm for 28 controls aged 11 ± 1 years with normal serum cholesterol (Jarvisalo et al. 2001), and 0.39 ± 0.03 mm for 48 controls aged 6 ± 3 years with normal cholesterol and triglyceride (Pauciullo et al. 1994). Recently, Ishizu et al. demonstrated that mean CIMT increased in a linear manner with age (estimated CIMT = [0.009 × age in years] + 0.35, r = 0.39, p = 0.002) from the data of 60 healthy children aged 5–14 years (Ishizu et al. 2004). In addition, our data indicated that the normal predicted value of CIMT was calculated in a linear regression equation (normal predicted CIMT = [0.004 × age in years] + 0.381, r = 0.61, p < 0.0001) derived from 35 healthy controls aged 19.6 ± 7.2 years (Noto et al. 2009). There was no significant difference between the normal predicted values of CIMT by Ishizu et al. and those in our study, even in a different age-cohort; however, the coefficients of these equations were entirely different. This implies that a nonlinear correlation should be assumed in the assessment of CIMT during childhood and adolescence. Following the establishment of age-specific reference values for CIMT by Jourdan et al., the reference ranges for subjects aged 6–18 years base on the measurements in 1155 Caucasian health controls have been published (Doyon et al. 2013). In the present study, we established the Asian reference values of CIMT at ages 6–20 years and also applied the Caucasian reference values of CIMT by Jourdan et al. because of the necessity of reference values at age 19–20 years. In fact, the present study including 12 articles features by far the largest adolescent population investigated. A cohort of subjects with a skewed age distribution like in this study should be assessed using valid age-related normative reference data.

Age-related increases in CIMT in healthy children do not appear to be strongly associated with pathological atherosclerotic progression. Indeed, increased CIMT may be related to intima or media hypertrophy or both, and may be an adaptive response to changes in flow, wall tension, or lumen diameter. Therefore, increased CIMT is not synonymous with atherosclerosis, particularly in the absence of carotid plaque. In the convalescent stage, even in transient coronary ectasia and regressed coronary lesions, intimal thickness has been detected by intravascular ultrasonography (IVUS) after KD (Mitani et al. 2009). In addition, increased arterial stiffness determined by pulse wave velocity occurred in KD patients with CALs (Cheung et al. 2007). Moreover, we have shown that observed higher CIMT may indicate the development of post-inflammatory arteriosclerotic remodeling after KD (Noto et al. 2012). These previous studies demonstrated that KD with CALs eventually led to the development of post-inflammatory arteriosclerotic remodeling characterized by luxuriant intimal proliferation and neoangiogenesis (Suzuki et al. 2000). On the other hand, it remains unclear whether the vessels in KD patients with CALs may more easily develop atherosclerotic changes. Recently, Chen et al. demonstrated pathophysiological findings on the marked acceleration of atherosclerosis following *Lactobacillus casei*-induced coronary vasculitis in a mouse model of KD (Chen et al. 2012). In a scientific statement from the American Heart Association’s expert panel, KD was listed among the eight pediatric diseases that are associated with a high risk of accelerated atherosclerosis in children (Kavey et al. 2006). Although there is no published data linking childhood CIMT measures with any clinical outcomes of interest for KD patients, longitudinal accurate CIMT measurements in individuals may provide a valuable tool for identifying and selecting candidates for early intervention, especially in KD patients with CALs.

Previous studies that used carotid ultrasound have been largely conflicting in regards to whether or not KD patients have a higher CIMT than normal controls. Although the exact reasons for these conflicting results remain unclear, differences in study populations, racial differences, methodology, pubertal status, latent coronary risk factors (CRFs), and systemic inflammation may play a role. In this study, the mean values of CIMT-SDS for N were more significantly different than those of CIMT-SDS for J between the KD patients and the controls among the 12 articles. Of note, compared with the normative references, controls were distributed within the normal range at a rate of 41.6 % for J and 91.6 % for N. These results indicate that not only meticulous selection of control subjects but also use of age- and race-specific reference values for CIMT are mandatory for performing accurate assessment of the vascular status in healthy children and adolescents and particularly in those after KD. Hence, we suspect that these conflicting results on CIMT in KD patients and controls may have been partially caused by the selection bias of healthy control subjects.

Several limitations might have influenced the results of this study. First, the sample size may be considered limited for constructing age-specific percentile curves and a reference table. The limited sample size may be compensated for by the absence of gender differences of CIMT. Second, we could not exclude healthy reference controls with latent CRFs including dyslipidemia and diabetes mellitus. Third, as the representative age for transformation, we applied not the individual ages but the mean age of the study population derived from each article, ignoring the age distribution of the subjects. Fourth, there is no published database focused on the childhood CIMT across race/ethnicity, age, and gender as yet. We could not perform accurate comparison across these variables between KD patients and controls. Finally, although the reproducibility of CIMT measurements should be strictly monitored to identify a subtle change of this parameter with age, we are unsure as to how the quality controls were being performed at each institute. These results should thus be interpreted with caution.

## Conclusions

Our results indicate that age and race-specific reference values for CIMT are mandatory for performing accurate assessment of the vascular status in healthy children and adolescents, particularly in those after KD. Future study with a large number of subjects per age group for the establishment of accurate reference values is warranted in children and adolescents considered at increased long-term cardiovascular risk.

## Abbreviations

CALs: coronary artery lesions
CIMT: carotid intima-media thickness
CRFs: coronary risk factors
KD: Kawasaki disease
LMS: least mean squares
SDS: standard deviation score
